# Heart Rate Correction of the J-to-Tpeak Interval

**DOI:** 10.1038/s41598-019-51491-4

**Published:** 2019-10-21

**Authors:** Katerina Hnatkova, Jose Vicente, Lars Johannesen, Christine Garnett, David G. Strauss, Norman Stockbridge, Marek Malik

**Affiliations:** 1 National Heart and Lung Institute, Imperial College, London, England; 2 Division of Cardiovascular and Renal Products, Office of New Drugs, Center for Drug Evaluation and Research, US Food and Drug Administration, Silver Spring, MD USA; 3 Division of Applied Regulatory Science, Office of Clinical Pharmacology, Office of Translational Sciences, Center for Drug Evaluation and Research, US Food and Drug Administration, Silver Spring, MD USA

**Keywords:** Cardiovascular biology, Risk factors, Engineering

## Abstract

Drug-induced changes of the J to T peak (JTp) and J to the median of area under the T wave (JT50) were reported to differentiate QT prolonging drugs that are predominant blockers of the delayed potassium rectifier current from those with multiple ion channel effects. Studies of drug-induced JTp/JT50 interval changes might therefore facilitate cardiac safety evaluation of new pharmaceuticals. It is not known whether formulas for QT heart rate correction are applicable to JTp and JT50 intervals. QT/RR, JTp/RR, and JT50/RR profiles were studied in 523 healthy subjects aged 33.5 ± 8.4 years (254 females). In individual subjects, 1,256 ± 220 electrocardiographic measurements of QT, JTp, and JT50 intervals were available including a 5-minute history of RR intervals preceding each measurement. Curvilinear, linear and log-linear regression models were used to characterize individual QT/RR, JTp/RR, and JT50/RR profiles both without and with correction for heart rate hysteresis. JTp/RR and JT50/RR hysteresis correction needs to be included but the generic universal correction for QT/RR hysteresis is also applicable to JTp/RR and JT50/RR profiles. Once this is incorporated, median regression coefficients of the investigated population suggest linear correction formulas JTpc = JTp + 0.150(1-RR) and JT50c = JT50 + 0.117(1-RR) where RR intervals of the underlying heart rate are hysteresis-corrected, and all measurements expressed in seconds. The established correction formulas can be proposed for future clinical pharmacology studies that show drug-induced heart rate changes of up to approximately 10 beats per minute.

## Introduction

The present regulatory guidance on drug-induced QT prolongation^1^ was highly successful in eliminating new pharmaceuticals with an increased propensity to trigger the Torsade de Pointes tachycardia. Since the inception of the guidance, no drug had to be withdrawn from the market because of this type of toxicity. Nevertheless, some potentially useful compounds that would not have been torsadegenic might have also been discontinued in their early development if they were found to block the delayed potassium rectifier current (IKr). Consequently, new strategies for assessing the implications of drug-induced QT prolongation are therefore presently under discussion^2–4^.

Among others, it has been observed that the QT interval prolongation might be separated into the prolongation of the interval between J point (end of the QRS complex) and the peak of the T wave (JTp interval) and the prolongation of the T peak to T end interval (Tpe interval) to differentiate drugs with different ion channel effects. In particular, three prospective clinical studies^5–7^ found that JTp interval prolongation (combined with Tpe prolongation) separate predominant IKr blockers from drugs that blocked not only IKr but also other cardiac ion channels that might mitigate the IKr blocking effects^8^. In more detail, these studies found that that if a QT-prolonging drug extends both JTp and Tpe intervals, it is likely a pure IKr blocker. On the contrary, if such a drug prolongs just the Tpe interval, its effects likely concern multiple ion channels that might alleviate the pro-arrhythmic effects of the IKr blockade. Hence, while the observation of drug-induced Tpe interval prolongation is not too informative in respect of the channel blocking mechanisms, observations of the presence or absence of drug-induced JTp interval are of regulatory interest.

For these reasons, it might be proposed to investigate drug-related JTp effects in addition to QT effects. To facilitate such investigations, heart rate correction of JTp intervals is needed. Similar to the heart rate correction of the QT interval^9^, it might be expected that if an investigated drug changes the heart rate profoundly, e.g. in excess of 10 beats per minute (bpm)^10^, subject-specific correction of JTp intervals or other methods reflecting the individuality of JTp/RR profiles would be needed. Nevertheless, in other cases, when the heart rate is changed only moderately (or not at all), some reasonable heart rate correction of JTp interval would still be needed in the same way as generic (e.g., Fridericia^11^) heart rate corrections are used for the QT interval. At the same time, it is not obvious whether generic QT correction formulas are applicable to the JTp interval.

Previous suggestion of JTp heart rate correction was based on relatively small numbers of drug-free ECG readings per subject and did not include the considerations of JTp/RR hysteresis^5,12^. Having this mind, we have investigated the JTp/RR patterns including the JTp/RR hysteresis profiles in a large population of healthy subjects with the aim of finding a reasonable generic correction formula. We also extended this investigation to the intervals between the J point and the median point of the area under the T wave (which we shall call the JT50 interval) which has also been observed to help separating drugs with different effects on myocardial ion channels^8^. For both the JTp and JT50 intervals, we compared their corrections to the corrections of the QT interval which we derived in the same way from the same population data.

## Methods

### Population and electrocardiographic recordings

Electrocardiogram (ECG) data analyzed in the study were obtained from two clinical pharmacology studies in healthy subjects. In these studies, repeated drug-free 12-lead Holter recordings were made in every participant. All subjects had normal screening ECG and normal clinical assessment^13^. Both original studies were approved by the relevant ethics boards (California Clinical Trials, Glendale; Spaulding Clinical Services, Milwaukee; Parexel International, Baltimore) and all participants gave written informed consent according to the Helsinki declaration. Because of the known effect of sleep on the QT interval duration^14^ which might be expected also for the JTp and JT50 intervals^15^, the present study used only day-time portions of the recordings.

Using previously published methods^16,17^, QRS onset, QRS offset (i.e., the J point), and T wave offset were made in multiple samples taken from the 12-lead Holter recording. The measurements were made in representative morphologies derived from 10-second ECG segments sampled at 1000 Hz. Previously reported pattern matching algorithms^18^ were also used to ensure that similar morphologies of the QRS onset and offset and of T wave offset were measured similarly. Quality control of the measurements included visual verification and manual correction of computerized measurements by at least two independently working cardiologists with data reconciliation in cases of their disagreement. Multiple measurements were made in each Holter recording while the subjects were in supine position and during free daily activity which included postural provocations. This led to substantial heart rate spans in each subject^17^. For each measured ECG sample, a 5-minute history of RR interval preceding the measurements was also obtained.

### T peak and T median measurements

The seminal studies^5–7^ that showed the usefulness of the assessment of drug-induced JTp changes converted the 12-lead ECG into orthogonal XYZ leads^19^ and applied a peak-detection algorithm^20^ to the vector magnitude of the orthogonal leads. As in the seminal studies, the 12-lead ECG recordings of the present study were obtained with Mason-Likar electrode configuration. The orthogonal conversion used in the seminal studies was designed for this electrode configuration and thus, the present study used the very same approach to the T peak (and thus JTp interval) definition. This approach was also found to provide most consistent within-subject comparisons of serial JTp measurements^21^.

In addition to the JTp interval, the area under the vector magnitude of the derived orthogonal leads was calculated between the J and T end points^22^. A median point was found dividing the area under the orthogonal vector magnitude into equal halves. The interval between the J point and this median point (the JT50 interval) was also considered since, as stated, it showed similar drug-classification properties as the JTp interval while showing lesser variability^8^.

**Figure 1:**
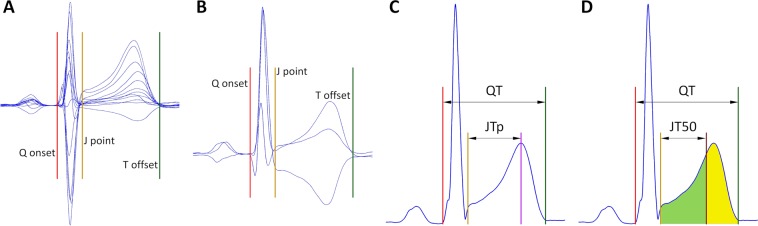
Schema of the measurement of ECG intervals evaluated in the study: Panel (A) shows all 12 leads of an ECG sample superimposed on the same isoelectric axis. Using images of this kind, QRS complex onset (red line), J point (i.e. QRS offset, amber line) and T wave offset (green line) are measured. Panel (B) shows the orthogonal XYZ leads derived from the original 12 leads. The originally made measurements of Q onset, J point and T offset are also shown superimposed on these orthogonal leads. Panel (C) shows the vector magnitude of the XYZ leads again with the Q onset, J point, and T offset measurements superimposed. The published algorithm^20^ is used to define the peak of the T wave (violet line). The QT interval is measured between the Q onset and T offset points and the JTp interval between the J point and the T wave peak. Panel (D) shows the same vector magnitude of the XYZ leads as shown in panel (C). T wave “middle” point T50 (brown line) is found such that it divides the area under the T wave vector magnitude (between J and T offset points) into equal halves. That is, the light-green and the yellow areas are of the same size. The JT50 interval is measured between the J point and the T50 point.

Figure 1 shows a schema of the QT, JTp, and JT50 interval measurements.

### Underlying heart rate measurements

For the heart rate correction of QT interval, the importance of QT/RR hysteresis has been well documented^23^. The QT interval does not depend on the instantaneously measured heart rate, so the speed of adaptation of the QT interval duration to the variable history of preceding heart rate needs to be taken into consideration^24,25^. The present study therefore investigated whether the same principle applies to the JTp and JT50 intervals. For the purposes of investigating this aspect, 4 different values of underlying heart rate (expressed by the corresponding RR intervals) were used:

#### Average of preceding 3 RR intervals

For each QT, JTp and JT50 measurement, the average of the 3 RR intervals preceding the measurement were obtained. Since the measurements were made on representative complexes of 10-second ECG segments, the average of 3 RR intervals at the end of the 10-second segment were used including a verification that the intervals in the corresponding heart beats did not differ from the representative complex measurements.

#### Average of 10-second RR intervals

The averaged RR interval was obtained from the 10-second ECG segment in which the measurement of the QT, JTp, and JT50 intervals was made.

#### Universal hysteresis correction

The 5-minute RR interval history of each interval QT, JTp and JT50 was processed with the previously published universal hysteresis correction^25^. As previously explained in detail^25^, the exponential decay model with a time-constant of 95% adaptation in 2 minutes was used to obtain a weighted average of the RR intervals preceding the QT, JTp, and JT50 measurement. This universal hysteresis correction approach was previously designed for the QT/RR analysis but for comparison, was used here for the JTp/RR and JT50/RR analysis.

#### Subject-specific hysteresis correction

For each of the study subjects and for each of the intervals QT, JTp, and JT50, each subject’s time constant was estimated for a single exponential decay model^24^. This profile provided subject-specific time-constants of the 95% adaptation (different for QT, JTp and JT50 interval in each subject) with which the weighted averages of RR intervals preceding the measurement were obtained. The subject-specific optimization of the hysteresis profiles was obtained by minimizing the residual of the curvilinear regression model to the underlying heart rate (see the next section on heart rate correction).

**Figure 2:**
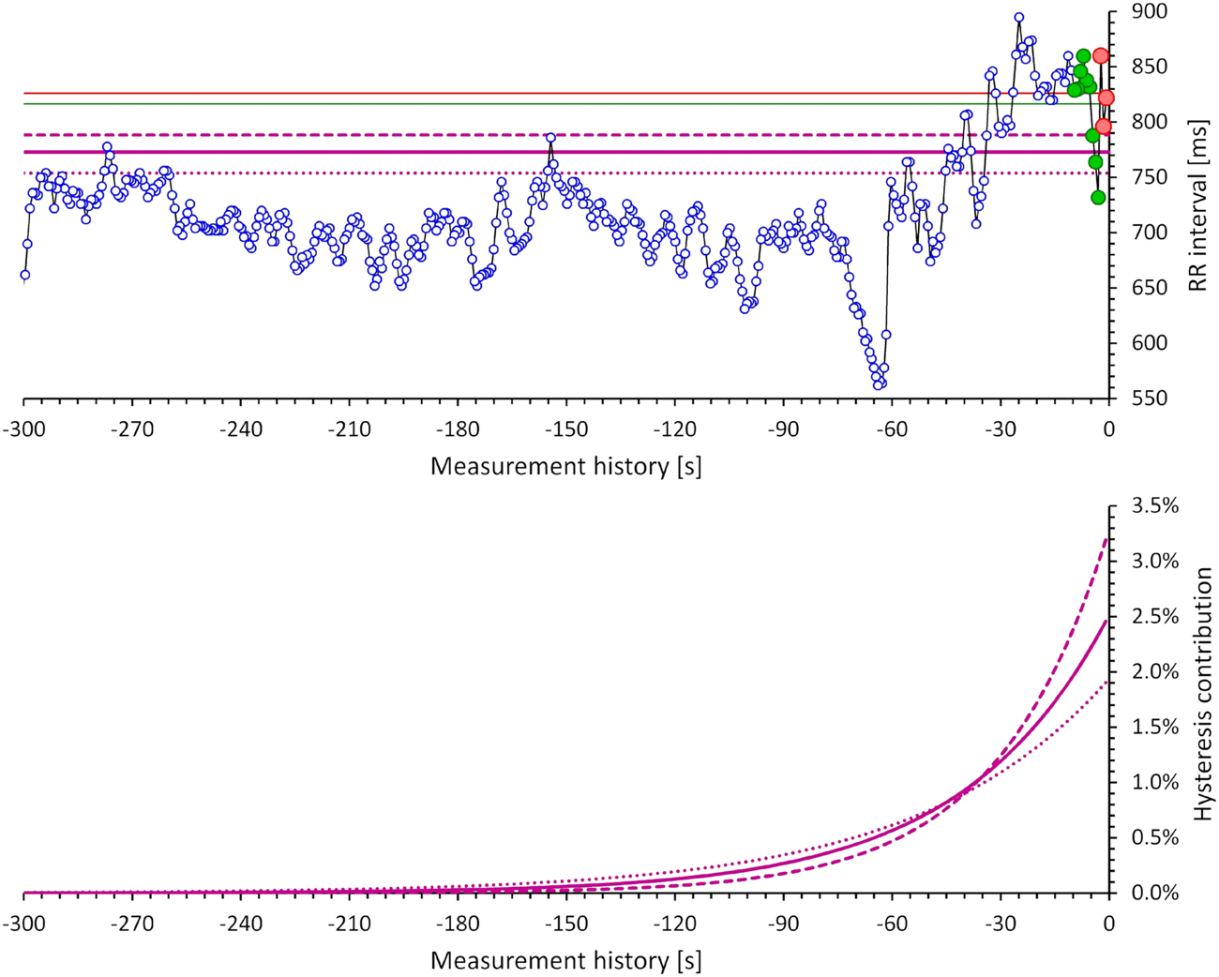
Schema of the different heart rate measurements: The *top panel* shows RR intervals in a 300-second history of ECG interval measurements (blue open circles with an interconnecting black line). That is, the interval measurements were derived from the ECG segment between −300 and 0 seconds of the horizontal time axis. The 3 RR interval immediately at the end of the measurement period are shown with red circles, the RR intervals within the 10 seconds of the measurement segment are shown with green circles. (See below for the explanation of the horizontal straight lines of the top panel). The *bottom panel* shows the weights used for weighed averaging the RR intervals in the history of the ECG measurements. The bold violet line corresponds to the exponential decay model of the universal correction with 95% adaptation after 2 minutes, the dashed and dotted violet line corresponds to exponential decay model with 95% adaptation after 1.5 and 2.5 minutes, respectively. When the universal hysteresis model is used or a subject-specific model is optimized, the series of the weights of the model is used to calculate a weighted average of the RR intervals in the history of the measurements (the integral of all the curves in the bottom panel is equal to 1). The straight horizontal lines on the top panel show the RR interval values corresponding to the heart rate expressions considered in the study. The red and green line shows the RR interval value corresponding to the average of the last 3 RR intervals and of the RR intervals in the 10 seconds of the measured ECG sample, respectively. The violet lines show the RR interval values obtained by the corresponding hysteresis models shown in the bottom panel.

Figure 2 shows a schema of the different heart rate measurements used in this study.

### Heart rate correction

For each study subject, for each the intervals QT, JTp, and JT50, and for each of the RR interval expressions defined in the previous section on heart rate measurements, three regression models were considered. In their definitions, all the intervals as well as all the RR interval definitions were considered in seconds.

#### Curvilinear regression model

As previously published^26^, curvilinear regression models appear to provide optimum subject-specific relationship between the QT intervals and the hysteresis corrected heart rate. Therefore, these models were also used for the JTp and JT50 intervals. The mathematical form of the models was ℐ_*i*_ = $\mu +\frac{\delta }{\gamma }(R{{R}_{i}}^{\gamma }-1)+{\varepsilon }_{i}$, where ℐ_*i*_ were the QT, JTp or JT50 intervals measured in the given subject, *RR*_*i*_ were the corresponding RR interval expressions (as defined in the section on underlying heart rate measurements), *ε*_*i*_ were normally distributed zero centered errors, and *μ*, *δ*, and *γ* were the subject-specific onset value at heart rate of 60 bpm, slope, and curvature of the regression.

This regression model leads to heart rate correction ℐ_*c*_ = ℐ $+\frac{\delta }{\gamma }(1-R{R}^{\gamma })$ and intra-subject residual of the model equals to the intra-subject standard deviation of the heart-rate corrected ℐ_*c*_ values.

#### Linear regression model

Intra-subject linear regression models were set-up in the mathematical form ℐ_*i*_ $=\,\mu +\beta (R{R}_{i}-1)+$ *ε*_*i*_ where ℐ_*i*_, *RR*_*i*_, *ε*_*i*_ and *μ* were as in the definition of the curvilinear regression model, and *β* was the linear slope of the ℐ/RR relationship. The mathematical form of the model corresponded to the Framingham^27^ or Schlamowitz^28^ QT corrections and, similarly to these corrections, it led to heart rate correction ℐ_*c*_ = ℐ $+\beta (RR-1)$. In each subject, the standard deviation of the heart-rate corrected ℐ_*c*_ values was again equal to the residual of the model.

In each subject, the regression parameter *β* was taken as the subject-specific correction coefficient of the linear model.

#### Log-linear regression model

Finally, intra-subject log-linear models were set-up in the form logℐ_*i*  _

$=\,\log \,\mu +\alpha \,\log \,R{R}_{i}+{\varepsilon }_{i}$, where ℐ_*i*_, *RR*_*i*_, *ε*_*i*_ and *μ* were again as in the curvilinear regression model definition and *α* was the slope of the log(ℐ)/log(RR) relationship. As in the Bazett^29^ or Fridericia^11^ correction formulas, this model led to the heart-rate correction in the form ℐ_*c*_ = ℐ $/R{R}^{\alpha }$. To compare the model with the other regression possibilities, the residual of the model in each study subject was defined as the within-subject standard deviation of the ℐ_*c*_ values. Note that contrary to the other models, this is different from the standard deviation of the *ε*_*i*_ values and reflects the true intra-subject variability of heart-rate corrected ℐ_*c*_ intervals.

In each subject, the regression parameter *α* was taken as the subject-specific correction coefficient of the log-linear model.

### Comparison of heart rate corrections

It was previously shown that incorporating QT/RR hysteresis into the heart-rate correction of QT interval increases the accuracy with which the QT interval is related to the underlying heart rate. In particular, it was shown that this increased accuracy reduced the intra-subject variability of QTc intervals^25^.

The same approach was used in this study. For both JTp and JT50 intervals (as well as, for comparison purposes, for the QT interval) a correction method (that is, a combination of a regression model with a RR-interval expression of the underlying heart rate) was considered significantly more accurate compared to a different correction method if it led to statistically significantly lower intra-subject regression residuals in the study population.

As explained with the individual regression model possibilities, the regression residuals were equal to the standard deviations of the *ε*_*i*_normally distributed errors. Since these errors were always zero centered, their standard deviation was a valid estimate of the sum of squares between the measured interval values and the regression model projections at the corresponding RR interval values. In other words, in each study subject, the regression residuals were measuring the closeness of the regression fit. Heart rate correction of an ECG interval ℐ is based on the concept^9,26^ that the optimum ℐ/RR regression model underlying the correction formula not only fits the drug-free ℐ/RR data without any bias (i.e. that the regression errors *ε*_*i*_ are zero centered) but that it also leads to the most compact regression with the lowest variability of the heart rate corrected ℐc values in drug-free measurements. The compactness of the regression and the variability of the ℐc values are represented by the variability of the *ε*_*i*_ errors. Hence, it is legitimate to use the regression residuals as the measure of the heart rate correction success and for the comparison of different regression models applied to drug-free data.

## Statistics

Data are presented as mean ± standard deviation. Within subject comparisons (e.g., of intra-subject regression residuals of different correction methods) were based on a paired two-tail t-test. Comparisons between female and male subjects of study population were based on a two-sample two-tail t-test assuming different variations. P-values below 0.05 were considered statistically significant. All statistical tests are shown and no correction for multiplicity was performed since the individual comparisons were not mutually independent.

## Results

### Population

Holter ECGs were recorded in 523 healthy subjects. Of these, 254 (48.6%) were females. A total of 236 (45.1%) and 259 (49.5%) study subjects identified themselves as of Black/African origin or White/Caucasians, respectively. The remainder classified themselves as Asian, Polar region natives, or “Other” race. At the time of the recording, female and male subjects were aged 33.4 ± 9.05 years and 33.7 ± 7.77 years, respectively (there were no statistically significant differences between ages of females and males).

### ECG data

A total of 660,657 ECG measurements of the QRS onset and offset and of T wave offset were made. Of these, 3,523 measurements (0.53%) were excluded because of substantial noise pollution within the JT interval. On average, 1,256 ± 220 non-excluded ECG measurements were available per subject (inter-quartile range 1,058–1,436).

The averaged, maximum, and minimum heart rates of the measured ECG samples in females were 76.4 ± 6.8, 120.4 ± 13.9, and 51.3 ± 6.2 bpm, respectively. The corresponding values in males were 71.7 ± 5.9, 115.8 ± 12.1, and 47.9 ± 5.6 bpm (p < 0.0001 for all three comparisons between females and males). The heart rate ranges, that is differences between maximum and minimum heart rates in measured ECG samples of individual subjects (i.e. the ranges available for the regression calculations) were 69.0 ± 13.9 and 67.9 ± 12.6 bpm in females and males, respectively (no statistically significant difference between the sex groups).

### Intra-subject curvilinear regression models

The results of the intra-subject curvilinear regression models combined with the subject-specific optimization of the heart-rate hysteresis time-constants are shown in Table 1. Most importantly, the residuals of the JTp/RR regressions were approximately 30% larger compared to those of the QT/RR regressions. On the contrary, the residuals of the JT50/RR regressions were marginally (but statistically significantly) lower than those of QT/RR regressions. Subject-specific time-constants of the JTp/RR hysteresis were similar to those of QT/RR hysteresis while those of the JT50/RR hysteresis were marginally smaller (i.e. the adaptation of JT50 intervals to heart rate was marginally faster). Table 1 also shows statistically significant differences in the curvatures of the profiles.

**Table 1:** Intra-subject curvilinear regression models.

	Mean ± Standard deviation		p-value F vs M	p-value vs QT (F)	p-value vs QT (M)
	Female	Male			
**QT**					
Onset [ms]	418.7 ± 13.3	398.7 ± 12.2	<0.0001		
Slope	0.159 ± 0.031	0.140 ± 0.024	<0.0001		
Curvature	0.619 ± 0.651	0.824 ± 0.712	0.0006		
Hysteresis [s]	111.9 ± 20.0	119.9 ± 22.2	<0.0001		
Residual [ms]	5.70 ± 1.12	5.50 ± 1.07	0.0318		
**JTp**					
Onset [ms]	232.4 ± 15.6	200.5 ± 14.4	<0.0001	<0.0001	<0.0001
Slope	0.157 ± 0.062	0.134 ± 0.075	0.0001	NS	NS
Curvature	0.453 ± 0.987	0.576 ± 1.085	NS	0.0030	<0.0001
Hysteresis [s]	129.5 ± 30.6	138.9 ± 32.7	0.0008	<0.0001	<0.0001
Residual [ms]	7.70 ± 2.35	6.80 ± 1.93	<0.0001	<0.0001	<0.0001
**JT50**					
Onset [ms]	204.2 ± 15.4	175.2 ± 12.6	<0.0001	<0.0001	<0.0001
Slope	0.133 ± 0.042	0.109 ± 0.047	<0.0001	<0.0001	<0.0001
Curvature	0.843 ± 0.992	1.139 ± 1.180	0.0019	<0.0001	<0.0001
Hysteresis [s]	100.0 ± 22.5	110.0 ± 26.3	<0.0001	<0.0001	<0.0001
Residual [ms]	5.27 ± 1.22	4.63 ± 1.02	<0.0001	<0.0001	<0.0001

The table shows the statistical summaries of the subject-specific parameters of curvilinear regression models involving RR intervals corresponding to the subject-specific individual hysteresis profiles (the hysteresis time constants of 95% adaptation are shown). For each parameter, a statistical comparison between female and male subjects (F vs M) is shown. For the parameters of JTp/RR and JT50/RR models, statistical comparisons (separate for female – F and male – M subjects) are shown for within-subject comparison with the corresponding parameters of the QT/RR models. As explained in the text, the regression onset values are presented at the heart rate of 60 beats per minute (i.e. the onset values shown in the table are the parameters *μ* of the curvilinear regressions).

### Variability of the linear and log-linear corrections

**Figure 3:**
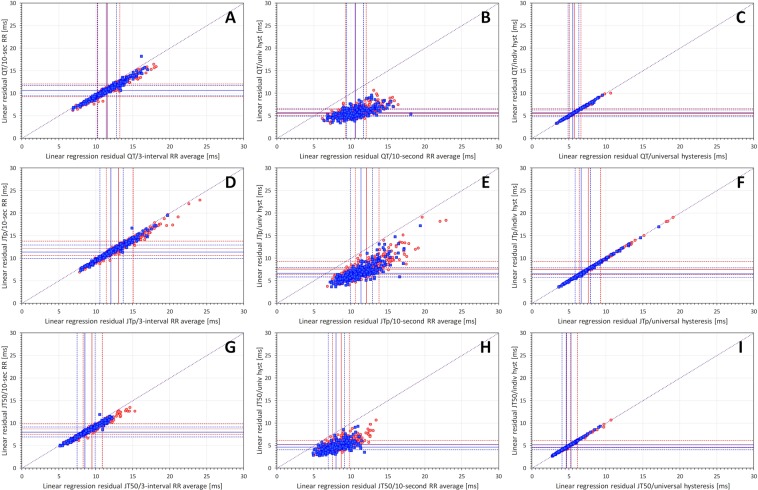
Comparisons of subject-specific linear regression residuals for different RR interval expressions. Left panels (A,D,G) show comparisons of residuals based on 3-interval RR averages with residuals based on 10-second RR averages. Middle panels (B,E,H) show comparisons of residuals based on 10-second RR averages with residuals based on universal heart rate hysteresis. Right panels (C,F,I) show comparisons of residuals based on universal heart rate hysteresis with residuals based on subject-specific individual heart rate hysteresis. Top line panels (A–C) show results for the QT/RR relationship; middle line panels (D–F) results for JTp/RR relationship; and bottom line panels (G–I) results for JT50/RR relationship. In each panel, red circles and blue squares correspond to female and male subjects, respectively. The full red and blue lines show the median residuals in women and men, respectively; the dashed red and blue lines show the inter-quartile ranges in women and men, respectively. In each panel, the dotted violet line shows the line of identity (note that in all comparisons, the marks of individual subjects are below this line). Univ – universal, indiv – individual, hyst – hysteresis.

Figure 3 shows that the accuracy of linear heart-rate corrections increases (a) when replacing the average of 3 RR intervals with the average of all RR intervals within the measured 10-second segment, (b) when replacing the 10-second RR average with the universal hysteresis correction, and (c) when replacing the universal hysteresis correction common to all subjects with the subject-specific hysteresis corrections. This principle applied equally to QT, JTp, and JT50 intervals. The Figure also shows that for all three types of ECG intervals, the largest reduction of the regression residuals among these three steps (that is the largest increase in the precision of the correction) occurs when replacing the 10-second RR average with the universal hysteresis correction. Finally, the Figure allows comparisons of the residuals of QT/RR, JTp/RR and JT50/RR regressions and shows the same difference as already observed with the curvilinear model. The variability of JTp/RR is noticeably larger than that of QT/RR and JT50/RR.

**Figure 4:**
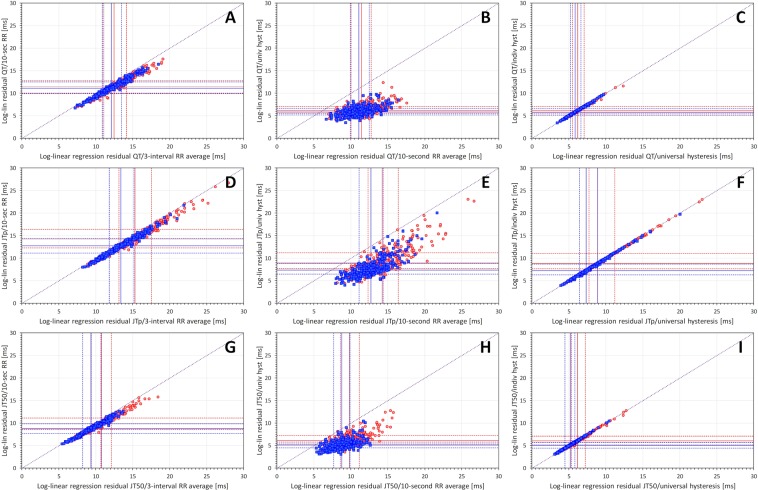
Comparison of log-linear residuals for different RR interval expressions. The layout of the figure is as in Fig. 3 only log-linear instead of linear residuals are shown. Log-lin – log-linear.

Figure 4 shows that the same relationships are valid for the log-linear regressions. Moreover, the comparison of Fig. 4 with Fig. 3 shows clearly that the variability of log-linearly corrected intervals is systematically larger than that of the linearly corrected intervals.

**Figure 5:**
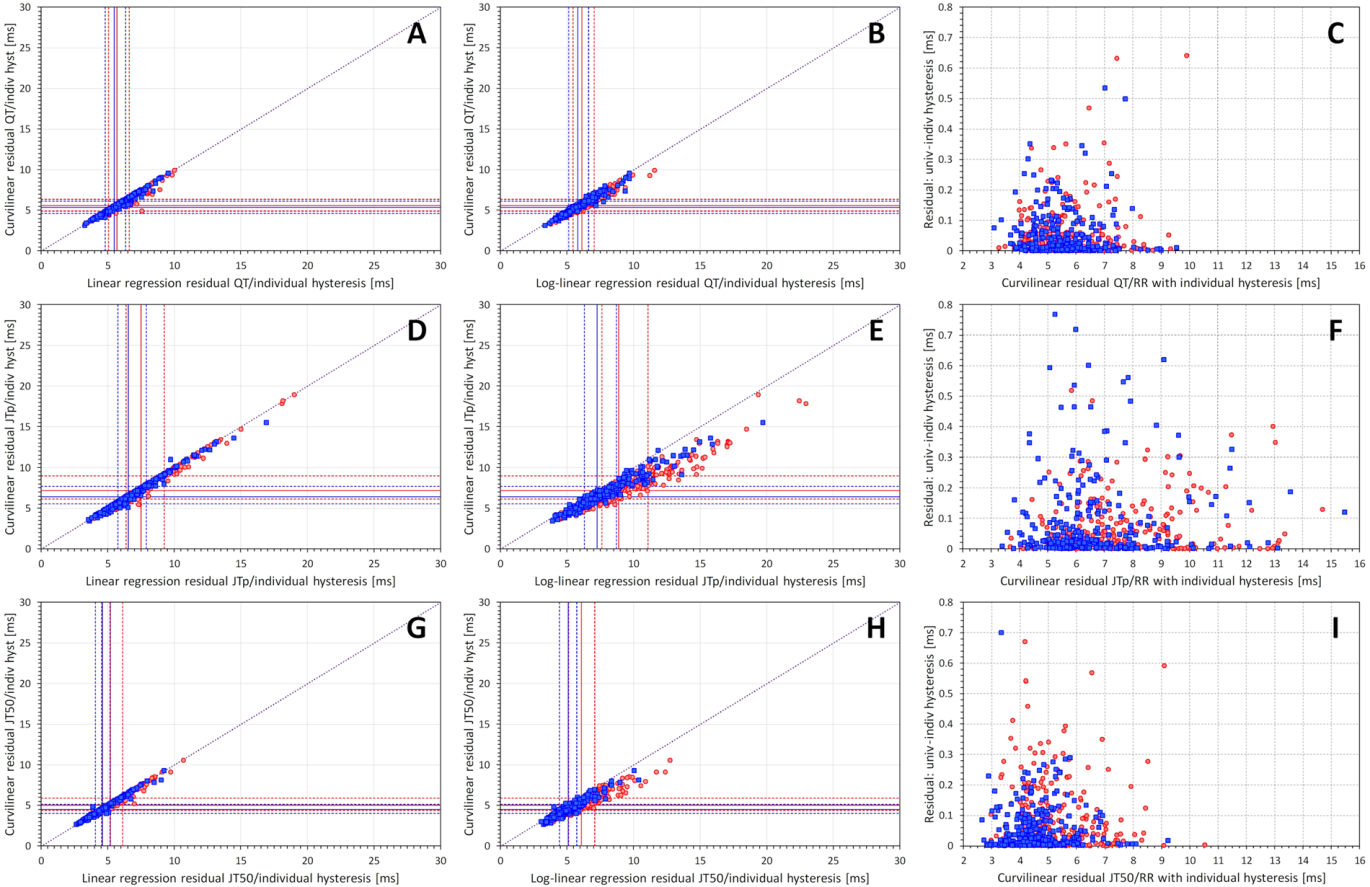
Panels (A,D,G) show comparisons of linear and curvilinear regression residuals both involving individual hysteresis. Panels (B,E,H) show comparisons of log-linear and curvilinear regression residuals both involving individual hysteresis (note that the reduction of residuals by curvilinear models is larger compared to the reduction from linear models). Panels (C,F,I) show the difference in residuals between curvilinear models using universal and individual hysteresis. Top line panels (A–C) show results for the QT/RR relationship; middle line panels (D–F) results for JTp/RR relationship; and bottom line panels (G–I) results for JT50/RR relationship. In all panels, red circles and blue squares correspond to female and male subjects, respectively. In the left and middle panels, the meaning of the red, blue and violet lines is the same as in Fig. 3. Univ – universal, indiv – individual, hyst – hysteresis.

Finally, Fig. 5 shows that whilst the use of curvilinear models reduces the variability of the corrected intervals compared to the linear and log-linear corrections, the reduction of the variability (albeit statistically significant) is not very large, particularly for the linear model. The rightmost panels of Fig. 5 also show that the step from universal hysteresis correction (common to all subjects) to individual subject-specific hysteresis correction is tiny. In all study subjects and for all three types of intervals (i.e., QT, JTp, and JT50), the introduction of the subject-specific hysteresis instead of the generic universal hysteresis reduced the variability of the corrected intervals by less than 1 ms.

### Linear and log-linear correction coefficients

The results described in the previous section show that similar to the QT interval, JTp and JT50 intervals do not depend on the instantaneously measured heart rate but that their heart rate adaptation is influenced by a prolonged history of preceding heart rate. That is, similarly to the QT interval, the JTp and JT50 intervals do not belong to the RR interval values obtained from the 3 preceding cardiac cycles or from the 10-second average because these do not reflect the full history of heart rate. Consequently, when using these RR interval values in the regression models, the slopes of the regressions get artificially shallower^30^ because introducing random noise into the independent variable of any linear regression reduces its slope.

**Figure 6:**
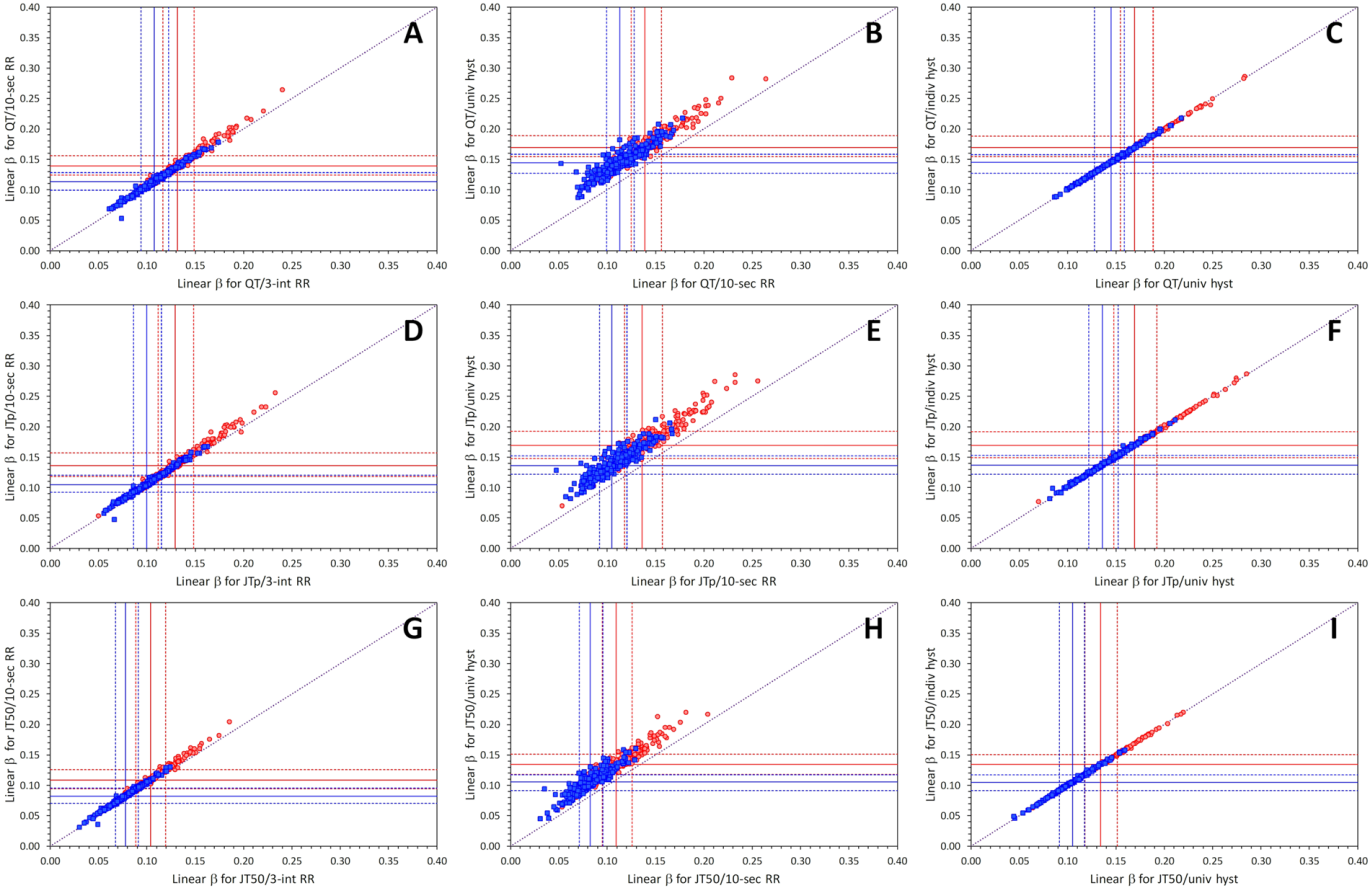
Comparison of subject-specific linear correction coefficients *β* for different RR interval expressions. The layout of the figure is the same as in Fig. 3 but instead of regression residuals, correction coefficients are shown. Compare with Fig. 3 and note that as the regression residuals decrease, the correction coefficients increase.

**Figure 7:**
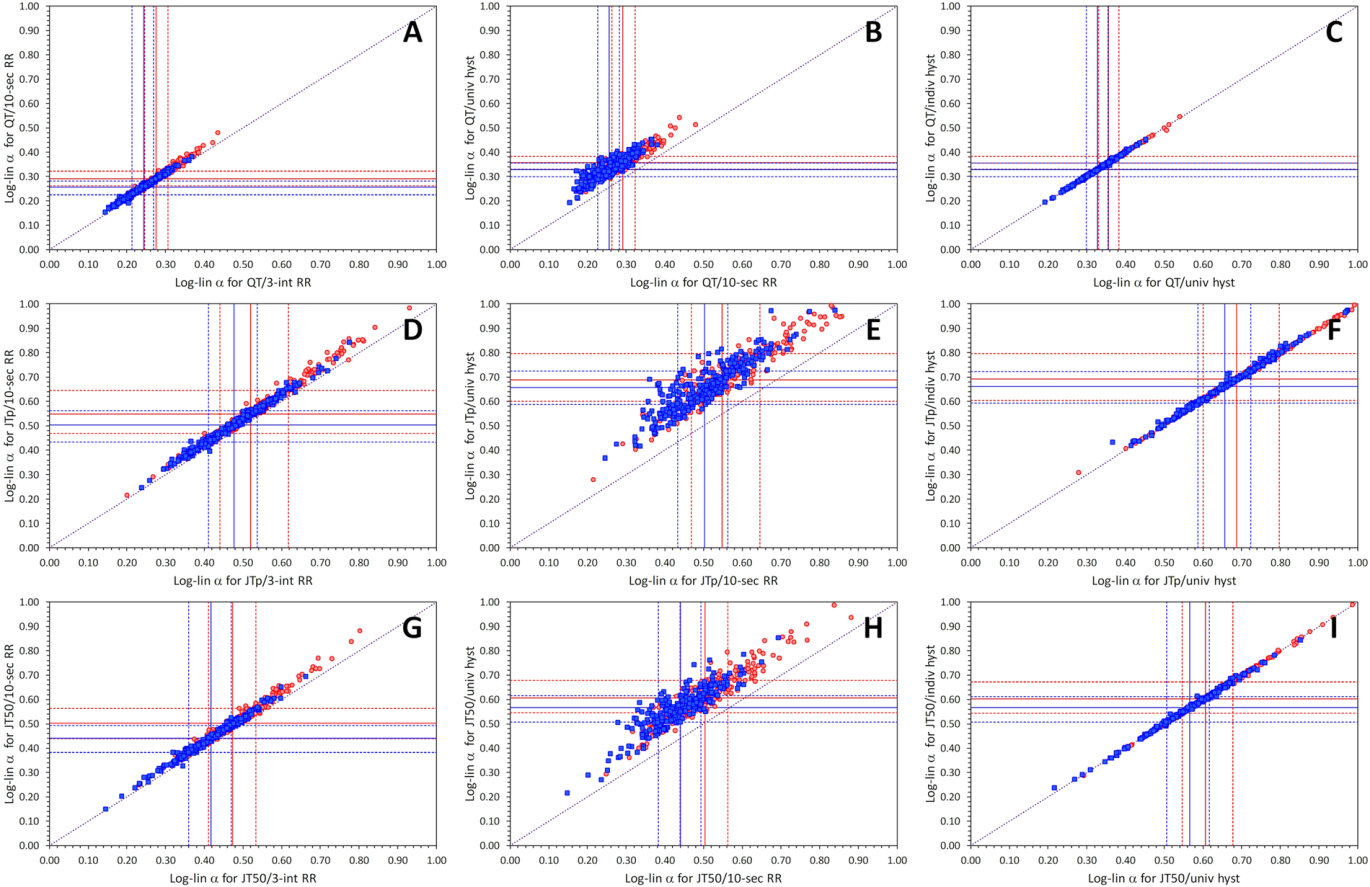
Comparison of subject-specific log-linear correction coefficients *α* for different RR interval expressions. The figure corresponds to Fig. 6 but instead of linear correction coefficients, log-linear coefficients are show. Compare with Fig. 4 and note that as the regression residuals decrease, the correction coefficients increase.

These effects on regression slopes (and on the corresponding heart-rate correction coefficients) are shown in Fig. 6 for the linear regressions and in Fig. 7 for log-linear regressions. The layout of the Figs 6 and 7 corresponds to the layout of Figs 3 and 4. The figures demonstrate that as the variability of the heart-rate corrected intervals decrease, the corresponding correction coefficients increase.

Comparison of Figs 6 and 7 also shows that whilst the mean linear correction coefficients are not very different for the QT/RR, JTp/RR, and JT50/RR regressions (Fig. 6), the mean log-linear correction coefficients of JTp/RR and JT50/RR patterns are substantially larger than those of QT/RR patterns. As previously explained^22^, this is for simple mathematical reasons related to the non-linear properties of the logarithmic transformation.

### Development of regression residuals and of correction coefficients

**Figure 8:**
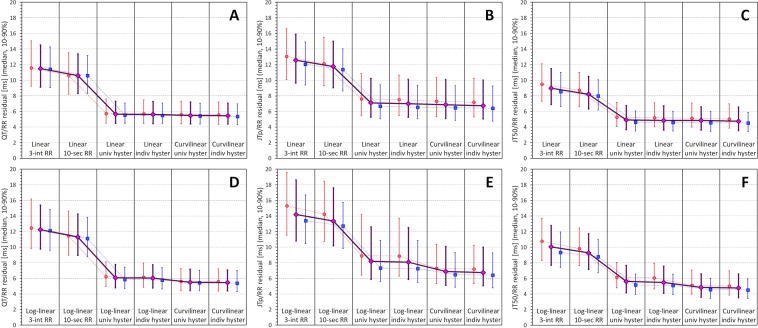
Summary of subject-specific linear and log-linear regression residuals. In each panel of the top line panels (A–C) the changes of linear regression residuals are shown for using 3-interval RR averages (3-int RR), 10-second RR averages (10-sec RR), universal heart rate hysteresis (univ hyster), and individual subject-specific heart rate hysteresis (indiv hyster). The last two sub-panels show the residuals of curvilinear regression models using universal and individual heart rate hysteresis. The bottom line panels panels (D–F) show the same for log-linear regression residuals (the last two sub-panels of each panel are the same in the top and the bottom line). Panels (A,D) correspond to QT/RR regressions, (panels (B,E) to JTp/RR regressions, and panels (C,F) to JT50/RR regressions. In each sub-panel, the red circles, blue squares and violet diamonds show the median values in women, men, and all subjects together, respectively. The error bars show the range between the 10^th^ and the 90^th^ percentile.

**Figure 9:**
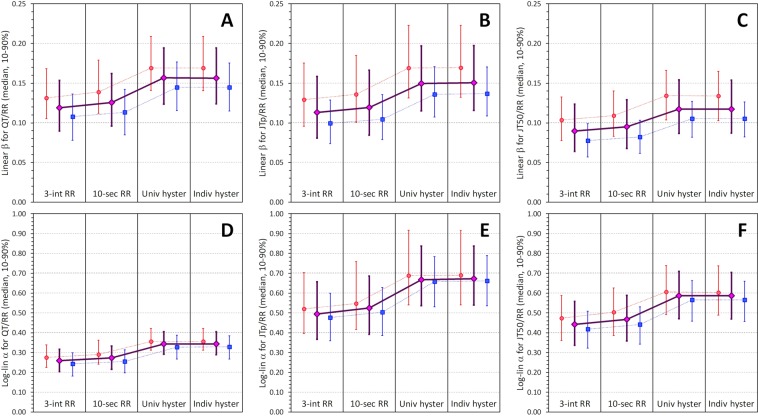
Summary of subject-specific linear and log-linear correction coefficients. In each panel of the top line panels (A–C) the changes of linear correction coefficients are shown for using 3-interval RR averages (3-int RR), 10-second RR averages (10-sec RR), universal heart rate hysteresis (Univ hyster), and individual subject-specific heart rate hysteresis (Indiv hyster). The layout of the bottom line is the same but log-linear correction coefficients are shown. The meaning of the symbols and error bars is the same as in Fig. 8.

Figure 8 shows the development of the regression residuals while Fig. 9 shows the development of linear and log-linear correction coefficients. Corresponding numerical data derived from the complete population are shown in Table 2.

**Table 2:** Regression residuals and correction coefficients.

RR interval expression	QT/RR		JTp/RR		JT50/RR	
	Residual [ms]	Correction coefficient	Residual [ms]	Correction coefficient	Residual [ms]	Correction coefficient
**Curvilinear regression model**						
Uni hysteresis	5.65 ± 1.11		7.31 ± 2.19		5.01 ± 1.17	
Indiv hysteresis	5.60 ± 1.10		7.24 ± 2.19		4.94 ± 1.17	
**Linear regression model**						
3 int RR average	11.69 ± 2.12	0.121 ± 0.027	12.74 ± 2.50	0.116 ± 0.030	9.14 ± 1.77	0.092 ± 0.024
10-sec RR average	10.71 ± 1.94	0.128 ± 0.028	11.91 ± 2.37	0.123 ± 0.032	8.39 ± 1.61	0.097 ± 0.025
Uni hysteresis	5.81 ± 1.16	0.159 ± 0.029	7.54 ± 2.21	0.154 ± 0.035	5.12 ± 1.20	0.120 ± 0.027
Indiv hysteresis	5.77 ± 1.15	0.159 ± 0.029	7.46 ± 2.20	0.155 ± 0.034	5.06 ± 1.20	0.119 ± 0.027
**Log-linear regression model**						
3 int RR average	12.43 ± 2.20	0.260 ± 0.047	14.54 ± 3.06	0.505 ± 0.113	10.18 ± 2.03	0.445 ± 0.090
10-sec RR average	11.41 ± 2.00	0.275 ± 0.049	13.71 ± 2.91	0.533 ± 0.119	9.40 ± 1.84	0.470 ± 0.095
Uni hysteresis	6.18 ± 1.21	0.344 ± 0.048	8.79 ± 2.85	0.680 ± 0.127	5.82 ± 1.45	0.587 ± 0.100
Indiv hysteresis	6.13 ± 1.20	0.344 ± 0.048	8.70 ± 2.85	0.683 ± 0.125	5.73 ± 1.44	0.585 ± 0.099

Table shows the regression residuals and linear and log-linear correction coefficients for different RR interval expressions in the complete study population. For comparison, regression residuals of the curvilinear regression coefficients are also shown. In within subject comparisons, all the values shown in the table were statistically significantly different, including those for which the presented rounded values are the same (e.g. QT/RR linear correction coefficients with universal and individual hysteresis were 0.158972 ± 0.029384 and 0.158797 ± 0.029196, respectively; p = 0.000285).

The largest reduction of the variability of heart-rate corrected intervals is seen with correction of heart-rate hysteresis. Since all the steps were statistically significant, the results of the study prove that heart-rate hysteresis needs to be considered not only for QT interval correction but also in studies of JTp or JT50 intervals.

### Generic JTp and JT50 heart-rate corrections

The population medians of correction coefficients might be proposed for generic corrections of JTp and JT50 intervals, to be used in the same way as Framingham^27^ and Fridericia^11^ corrections are used, are appropriate in QT studies in which the heart rate changes are not substantial^9^.

As discussed further, again similar to generic QT correction, it appears fully appropriate to combine the following correction with the universal heart-rate hysteresis correction that corresponds to the 2-minute time-constant of 95% interval adaptation^25^. In the following formulas, the symbol $\overline{{\rm{RR}}}$ means a weighted average of preceding RR intervals calculated according to the previously published formula for generic hysteresis correction^25^ and expressed in seconds.

#### Generic linear-correction

Using the median values of the population, the following formulas (without the sex distinction) can be proposed: ${\rm{JTp}}c={\rm{JTp}}+0.150(1-\overline{{\rm{RR}}})$ and ${\rm{JT}}50c={\rm{JT}}50+0.117(1-\overline{{\rm{RR}}})$.

Alternatively, the following formulas might be used in women: ${\rm{JTp}}c={\rm{JTp}}+0.169(1-\overline{{\rm{RR}}})$ and ${\rm{JT}}50c={\rm{JT}}50+0.134(1-\overline{{\rm{RR}}})$ while in men, these formulas appear appropriate: ${\rm{JTp}}c={\rm{JTp}}+0.136(1-\overline{{\rm{RR}}})$ and ${\rm{JT}}50c={\rm{JT}}50+0.105(1-\overline{{\rm{RR}}})$.

When applying these generic corrections to the study population, the following intra-subject standard deviations were found. JTpc: 8.59 ± 2.61 ms in the total population; 8.99 ± 2.75 ms in women separately, and 7.77 ± 2.09 ms in men separately (p < 0.0001 for the sex difference). JT50c: 6.07 ± 1.68 ms in the total population; 6.22 ± 1.66 ms in women separately, and 5.39 ± 1.31 ms in men separately (p < 0.0001 for the sex difference).

Importantly, these intra-subject standard deviations of the heart-rate corrected intervals were significantly lower than the standard deviations of the intervals corrected with subject-specific corrections based on the averages of the 3 RR intervals or the 10-second averages of RR intervals (compare the values with Table 2; p < 0.0001 for all comparisons).

When the same principle was applied to the QT interval data of the complete population, a general correction coefficient of *β* = 0.157 was found, which is very close to the Framingham formula^27^.

#### Generic log-linear correction

As discussed further, the log-linear correction appears significantly inferior to the linear correction. Nevertheless, again using the median values of the correction coefficients in the study population, the following formulas were obtained: ${\rm{JTp}}c={\rm{JTp}}/{\overline{{\rm{RR}}}}^{0.667}$ and ${\rm{JT}}50c={\rm{JT}}50/{\overline{{\rm{RR}}}}^{0.587}$.

Sex-specific variants of these corrections are: ${\rm{JTp}}c={\rm{JTp}}/{\overline{{\rm{RR}}}}^{0.688}$ and ${\rm{JT}}50c={\rm{JT}}50/{\overline{{\rm{RR}}}}^{0.607}$ for women, and ${\rm{JTp}}c={\rm{JTp}}/{\overline{{\rm{RR}}}}^{0.657}$ and ${\rm{JT}}50c={\rm{JT}}50/{\overline{{\rm{RR}}}}^{0.566}$ for men.

When applying these generic corrections to the study population, the following intra-subject standard deviations were found. JTpc: 9.58 ± 3.11 ms in the total population; 10.69 ± 3.34 ms in women separately, and 8.55 ± 2.44 ms in men separately (p < 0.0001 for the sex difference). JT50c: 6.40 ± 1.75 ms in the total population; 7.01 ± 1.84 ms in women separately, and 5.77 ± 1.40 ms in men separately (p < 0.0001 for the sex difference).

Again, these standard deviations were statistically significantly smaller than those achieved with subject-specific corrections based on the RR interval expressions that did not involve heart rate hysteresis (p < 0.0001 for all). Nevertheless, these standard deviations were also statistically significantly larger than those shown in the previous section on generic linear corrections (again p < 0.0001 for all comparisons).

For the QT data of the complete population, the median log-linear correction coefficient *α* = 0.343 was very close to the Fridericia correction^11^.

## Discussion

The study provides four principal conclusions.

Firstly, based on the analysis of a reasonably sized population of healthy subjects, a proposal of generic heart-rate corrections for the JTp and JT50 intervals was possible. Although we also report sex-specific variants of the formulas, it should be noted that the improvement of the intra-subject standard deviations of JTpc and JT50c was modest when adopting the sex-specific generic corrections. Since every generic correction formula is inherently polluted by some errors due to the inter-subject variability of the JTp/RR and JT50/RR patterns (see the numerical values in Table 2), the use of the sex-independent formulas might be proposed for future studies in which the heart rate does not show substantial changes.

Secondly, the study confirms that, similar to the QT interval duration, the JTp and JT50 intervals are dependent on the history of the underlying heart rate rather than on the instantaneous heart rate taken at the time of the interval measurement. This is not surprising since there is an obvious physiologic relationship between the QT, JTp and JT50 intervals^22^. The incorporation of the heart rate hysteresis into the correction of the JTp and JT50 intervals has a profound effect on the accuracy of the correction. As we have shown, generic universal correction formulas combined with generic universal correction of heart rate hysteresis is significantly more accurate than subject-specific modeling of JTp/RR or JT50/RR patterns that do not incorporate the hysteresis effects.

Thirdly, the comparison of the variability of rate corrected JTp and JT50 intervals shows that the JT50 measurement is significantly more precise. Thus, if the previous observations that JT50 serves equally well in the classification of drugs to predominant IKr versus multichannel blockers^8^, the JT50 interval would clearly be preferred. The measurement of the JT50 interval also does not suffer from the potential problems of T peak definition that might occur in strangely formed ECG patterns^31^.

Finally, with both JTp and JT50 intervals, the study shows that the linear correction formulas are significantly more accurate than the log-linear formulas. This is again consistent with what has been previously described in relation to the individual QT/RR patterns^32^.

The generic JTpc and JT50c formulas that we propose will naturally suffer from the same problem as generic QTc formulas^9^. They should not be used with every level of underlying heart rate change. Once an investigated drug changes heart rate more than moderately, subject-specific profiles of drug-free JTp/RR and JT50/RR patterns need to be established based on interval measurements over a wide heart rate span^9^. The dependency of the imprecision of the proposed JTpc and JT50c formulas on the underlying heart rate changes needs to be further investigated. Nevertheless, pending such an investigation, similar approach as presently applied to the QTc intervals might perhaps be considered reasonable. We have recently reported that Fridericia formula is reasonable in pharmacological studies if observed drug-induced heart rate changes do not exceed 10 bpm and that combination of Fridericia formula with universal correction for QT/RR hysteresis substantially improves QTc data accuracy^10^. We believe that we can reasonably speculate that a similar limit of heart rate changes would also apply to JTpc and JT50c intervals derived by the proposed universal formulas.

Whilst the JTp/RR and JT50/RR hysteresis show inter-individual differences (see Table 2) and while the heart-rate adaptation of the JTp and JT50 intervals was significantly slower and faster, respectively, compared to that of the QT interval, the observed differences were modest. Therefore, the application of universal generic hysteresis previously proposed for the QT interval correction^25^ appears practical. Incorporating this hysteresis requires no more than finding the 5-minute history of individual RR intervals preceding the interval measurements. Since a vast majority of clinical pharmacology studies uses continuous Holter recordings and since computerized identification of QRS complexes should not present any substantial problems in digital ECG data^33^, correction for heart rate hysteresis does not complicate the ECG evaluations of clinical studies.

In addition to the accuracy differences between the JTp and JT50 intervals, the study also observed lower slopes of the JT50/RR patterns compared to the JTp/RR patterns. The JTp/RR patterns were also found more curved. This likely corresponds to the known shifts towards more symmetrical T waves during fast rates.

Intentionally, the presentation of the study results omitted some of the combinations of correction regressions and RR interval expressions. The application of the curvilinear model^26^ requires convergence of curvature searches that is potentially problematic if the RR interval data are polluted by substantial noise such as with the 3-interval or 10-second averages. Along the same line, some of the presented results are illogical. For instance, a combination of the linear model with individually optimized hysteresis profile makes little sense since the hysteresis optimization requires the more accurate curvilinear model to be established^17^. None of these aspects apply to the combination of the generic formulas with the generic universal hysteresis correction since this combination does not require to study individual drug free JTp/RR or JT50/RR (or QT/RR) profiles.

In addition to the lower variability of the heart-rate corrected intervals, the linear correction also does not suffer from the transformation problems that complicate the log-linear corrections^22^. Even with the QT interval, there are no physiologic, methodologic, or mathematical reasons to prefer the correction formula in the form of QT/RR^α^. Not only is the coefficient α influenced by the mean duration of the corrected intervals (note the very substantial exponent difference between the Fridericia formula and the JTpc correction as proposed here), but it also has awkward non-additive properties^9^. The popularity of this correction form, possibly triggered by the initial work on the individual subject-specific corrections^34^, is thus entirely unjustified^32^.

The previously proposed correction of the JTp interval^5^ used only log-linear model and derived a correction coefficient of 0.58. This was based on data from studies which included fewer drug-free ECG measurements and for which no heart rate history (needed for the assessment of heart-rate hysteresis) was available. Consequently, the previously derived coefficient was between the results presented here for the 10-second RR average and the universal hysteresis correction. As far as we are aware, comparisons of heart rate corrections of JTp and JT50 intervals has not been investigated before.

### Limitations

The position of the peak of the T wave is known to depend on the ECG lead^30^. Therefore, consistent with the seminal investigations of the drug classification^5–7^ this study used the vector magnitude of orthogonal XYZ leads to represent the T wave signal in one dimension^21^. There are many other possibilities of representing the multi-lead T wave signal. While it is unlikely that the results would be very different with other T wave representations, they were not researched in this study. The source data were obtained in healthy volunteers of relatively young age. Although the population was reasonably representative of subjects that would be investigated in other clinical pharmacology studies, no comment can be made on whether the results apply also to elderly subjects or to patients with ECG abnormalities. In addition to the linear and log-linear models, other regression forms have also been proposed to study the QT/RR relationship^35–37^. Such regressions forms were not investigated. Similarly, only the exponential decay model of hysteresis correction was used^24,25^ while other matheatical possibilities have previously been proposed^23^. Since the differences among the universal and subject-specific hysteresis corrections were modest, it is unlikely that other hysteresis expressions would lead to different conclusions.

### Practical implications

Despite the study limitations, it seems reasonable to propose that the established formulas for correction the JTp and JT50 intervals for the underlying heart rate and heart rate hysteresis should be used in future clinical pharmacology studies in which the drug-related changes of these intervals are investigated and in which no substantial heart rate changes are observed. Further investigations are needed on the JT50 interval used in the classification of drug effects. If the usefulness of this measurement were confirmed, replacing JTp with JT50 may improve the accuracy of clinical investigations. Unless good reasons are shown for using the log-linear correction, linear correction of JTp and JT50 for the underlying heart rate should be preferred.

We envisage the use of these formulas in clinical pharmacology studies of drugs found to prolong the QTc interval. The vast majority of such studies use continuous 12-lead Holter recordings and the measurement approach that we have described should be easily implemented without any substantial additional procedures. As we explained in the Introduction, establishing the absence or presence of drug-induced JTpc or JT50c prolongation is a valid addition for a regulatory judgement of a QTc prolonging compound.
